# COVID-19 and Power in Global Health

**DOI:** 10.34172/ijhpm.2020.72

**Published:** 2020-05-06

**Authors:** Amy Patterson, Mary A. Clark

**Affiliations:** ^1^Department of Politics, University of the South, Sewanee, TN, USA.; ^2^Department of Political Science, Tulane University, New Orleans, LA, USA.

**Keywords:** COVID-19, Power Relations, Issue Framing, Pandemic Response, Health Disparities

## Abstract

Political scientists bring important tools to the analysis of the coronavirus disease 2019 (COVID-19) pandemic, particularly a focus on the crucial role of power in global health politics. We delineate different kinds of power at play during the COVID-19 crisis, showing how a dearth of compulsory, institutional, and epistemic power undermined global cooperation and fueled the pandemic, with its significant loss to human life and huge economic toll. Through the pandemic response, productive and structural power became apparent, as issue frames stressing security and then preserving livelihoods overwhelmed public health and human rights considerations. Structural power rooted in economic inequalities between and within countries conditioned responses and shaped vulnerabilities, as the crisis threatened to deepen power imbalances along multiple lines. Calls for global health security will surely take on a new urgency in the aftermath of the pandemic and the forms of power delineated here will shape their outcome.

The coronavirus disease 2019 (COVID-19) pandemic has wrought enormous loss of human life and depressed economic growth worldwide. The containment of the virus and treatment of its victims overshadows all other concerns as intensive care unit beds are requisitioned, personnel shifted, budgets repurposed, supplies redirected, and governments scramble to address the crisis. Political scientists bring important tools to the analysis of the pandemic, particularly a reflection on the crucial role and the various forms of power in health.^1^ The weakness of compulsory, institutional, and epistemic power sheds light on the emergence of the COVID-19 pandemic, while the pandemic itself has laid bare the ways that productive and structural power not only affect responses but are also reproduced through the crisis.

Multiple, overlapping forms of power are evident in global health, a fact that shapes global health policy and advocacy.^2^ Power can be coercive, as in ‘A has power over B to the extent that he can get B to do something that B would not otherwise do.’^3^ An overabundance of such compulsory power (often rooted in material resources) can skew global health priorities.^4^ Institutional power is embedded in informal and formal rules and decision-making bodies like the World Health Organization (WHO). Until its decline in the 1990s, the WHO’s power reflected its ability to coordinate the views of health experts, its representation from most countries in the world (all with equal voting rights), and its financial resources.^5^ The WHO has some epistemic power, or the ability to speak authoritatively because of its expertise; however, if we examine global health more broadly, the field lacks epistemic power because of its multidisciplinary and interdisciplinary nature. Despite how its ‘claims to knowledge’ may be portrayed as ‘objective truth’ rooted in ‘neutral’ evidence,^6^ global health lacks a common epistemology, sense of causal beliefs, and methodology.^4^

Normative power is apparent when some have legitimacy and influence because they claim to speak for ethical principles, such as WHO’s advocacy for a ‘right to health for all.’ At the national level, this power is embodied in a social contract, whereby citizens consent to government engagement on population health. At the global level, no normative social contract exists, and the exercise of power can lack legitimacy because it does not rest on inclusive forms of consultation with the people it most affects.^5,7^ Structural power manifests itself in economic, social and/or political relationships that reinforce subjective interests, while productive power undergirds social processes that create meaning. The ‘unconscious and unacknowledged nature’ of these forms of power perpetuates health disparities,^8^ and it enables those who benefit from these forms of power to decide ‘what does and does not get taken seriously.’^9^

The emergence of the COVID-19 pandemic demonstrates the limited scope of compulsory and institutional power. Even though powerful countries (China, the United States) were affected first, they were unable to compel other countries to act to curtail the virus. For example, Italy could not make European Union countries share health supplies, and the United States could not force China to share data on early cases. Similarly, because the WHO has no enforcement mechanism to punish its members for health actions (or inactions) and thus, must rely on diplomacy, it could not make countries avoid travel bans, share case counts, or do early testing. Similarly, the WHO’s institutional power was limited, despite the fact that the International Health Regulations obligate countries to notify the WHO about emergent health threats to facilitate a coordinated international response. With the force of international law, the International Health Regulations, modified after China delayed disclosure of the severe acute respiratory syndrome (SARS) outbreak in 2002-2003, cover novel infectious diseases and authorize the WHO to declare an international health emergency.^10^ However, because the WHO depends on countries to recognize and respond to its institutional power, it is at the mercy of political processes. Chronic budget shortfalls, the rise of other health-focused bodies like the Gates Foundation, financial dependence on wealthy countries, and what are often perceived to be non-inclusive decision-making processes also undermine the WHO’s institutional and normative power.^5^

Similarly, as Grépin might predict,^4^ global health’s challenge in fostering coherent, epistemic power meant that early, effective responses rooted in scientific and public health expertise lagged and infections soared. Public health experts struggled to assert the superiority of their tools for preventing, diagnosing, and treating COVID-19 against political leaders who questioned their authority and seemed to espouse a non-traditional source of epistemic power. Scientific studies of the virus’s genome and development of accurate diagnostic tests were pitted against populist disregard of the virus’s severity or blind faith in untested treatments. While most world leaders championed the scientific approach (eg, German chancellor Angela Merkel), others exhibited a dim view of traditional scientific epistemology. US President Trump effectively proposed dropping current guidelines for drug trials by demanding that hydroxychloroquine be tested on humans as a possible treatment for COVID-19. Similarly, presidents Jair Bolsonaro (Brazil), Daniel Ortega (Nicaragua), and Alexander Lukashenko (Belarus) downplayed the virus’s seriousness and resisted expert advice on social distancing, actions that facilitated viral spread.^11^

Productive power undergirds how policy-makers and publics framed the pandemic and then responded. As discursive tools, frames enable individuals to ‘simplify and make sense of the world around them,’^12^ and they ‘determine automatically and repetitiously’ subjective perceptions of reality.^8^ They can galvanize action, particularly when they manifest frame coherence, or consensus among experts about the problem and its solutions, and when that consensus resonates with the public and policy-makers.^12^ Initially, leaders framed COVID-19 as an existential threat to national security, with the response being a ‘war,’ the virus being an ‘enemy,’ and healthcare workers termed ‘warriors.’ This frame mobilized publics for behaviors like social distancing. But it also enabled some governments to justify human rights abuses, and it contributed to panic-buying, stock sell-offs, risking healthcare workers’ lives without adequate protective equipment, and demonizing and scapegoating people believed to carry the virus like Asian-Americans.^13^ These processes deepened the productive power of racism and fear in making social meaning.

As the virus spread globally, an economic cost-benefit frame took hold. Lockdowns, market closures, and the resulting unemployment threatened livelihoods for millions, making the economic cost of curbing the virus seem too high. This frame resonated with policy-makers, and countries like Ghana, the United States, and Germany began to relax population movement restrictions, even though public health officials warned about a resurgence of cases. (This also showed the limits of epistemic power). A few leaders espoused a human rights frame that stressed the right to testing, ventilators, and dignified care, as well as solidarity to protect the most vulnerable people. This frame embodied a normative power that may check other forms of power that harm health.^6^ Yet without the backing of compulsory or institutional power from a powerful country, this frame did little more than motivate community action. Frame incoherence on COVID-19 made global collaboration difficult, a pattern evident on other issues like mental health and early childhood development.^14^

Both responses to COVID-19 and the pandemic’s economic impact elucidate structural power inequalities among diverse countries. As an example, countries with substantial industrial bases increased production of masks, respirators, and other medical supplies, although reliance on just-in-time global supply chains hampered re-tooling. Countries with little industrial capacity, in contrast, had less ability to protect and treat their citizens, and thus, had to depend on donor aid. Because many people in the global South depend on wages from unskilled labor, informal-sector work, or migrant remittances and lack access to savings and publicly funded social safety nets, the pandemic-induced economic recession may be more devastating to them than to populations in the global North. Hoarding and export restrictions have caused price surges for wheat and rice, two global food staples,^15^ with probable knock-on effects of greater poverty-induced malnutrition, disease, and bodily insecurity. To address the pandemic’s economic fallout, the International Monetary Fund (IMF) committed to assisting countries with high debt burdens and providing poor countries with emergency reserves, a proposal the United States blocked in order to restrict benefits to Iran and Venezuela.^16^ Structural power imbalances were reproduced, as poor countries had to turn to the IMF, and the United States could capitalize on this dependence to pursue its geopolitical goals. Structural power intertwined with institutional power, as the United States used its IMF voting rights (determined by a country’s contribution size, which is based on its economy) to sway decisions.

Within countries, structural power rooted in the long-term accumulation of socioeconomic advantage and disadvantage along race, class, and other lines underlies the unequal impact of COVID-19 on people’s health, ability to protect themselves from infection, and economic wellbeing. For example, African Americans have suffered a disproportionate number of deaths, an outcome that has been linked to long-standing racial disparities in living environments and inadequate care for chronic diseases. Structural power overlaps with the productive power of racism, which fosters measurable stress on bodies and undermines patient-physician trust.^17^ In addition, structural power is apparent in that some individuals—healthcare workers, grocery store clerks, restaurant staff—must do their job in person, putting them both at risk of exposure and, for some, risk of unemployment in a lockdown. Increased unemployment deepens structural imbalances, as those with smaller savings and fewer assets struggle to ride out the economic downturn. Low-income people are disproportionately affected by school closings, which can depress learning (particularly with no online options), lower parents’ productivity, harm child nutrition (without school feedings), and increase the risk of violence toward children.^18^ Educational setbacks deepen power imbalances and economic inequities.

In conclusion, the weakness of compulsory, institutional, and epistemic power explains the failure of the WHO and individual countries to stop COVID-19, while productive and structural power undergird responses. The pandemic initially appears to have given proponents of isolationism and unilateralism the upper hand as they argue that the economic and human costs of COVID-19 prove global health governance’s failure. But because forms of power lie in tension,^1,2^ this is not the only alternative. Motivated by the pandemic’s high cost to global commerce and the loss of normative leadership in human development, powerful countries may exercise compulsory or institutional power to strengthen the WHO, improve international infectious disease surveillance, and assist poor countries in building stronger health systems. On-going research about the virus and the outcomes of public health measures could deepen the epistemic power of proponents of reducing health disparities and bolster global health cooperation. While all parties may emerge from the pandemic calling for improved health security, whose vision will prevail depends on the elements of power discussed here.

## Ethical issues

Not applicable.

## Competing interests

Authors declare that they have no competing interests.

## Authors’ contributions

Both authors conceptualized, wrote, edited, and revised the manuscript.

## Authors’ affiliations

^1^Department of Politics, University of the South, Sewanee, TN, USA. ^2^Department of Political Science, Tulane University, New Orleans, LA, USA.
